# Effects of variable-temperature heat reservoirs on performance of irreversible Carnot refrigerator with heat recovery

**DOI:** 10.1038/s41598-023-50011-9

**Published:** 2023-12-27

**Authors:** Zhe Zhang, Huan Su, Guoqiang Dai, Xiaohua Li, Liping Zeng

**Affiliations:** 1 Department of Building Engineering, Hunan Institute of Engineering, Xiangtan, 411101 China; 2 Hunan Engineering Research Center of Energy Saving and Material Technology of Green and Low Carbon Building, Xiangtan, 411104 China

**Keywords:** Energy science and technology, Engineering

## Abstract

The outlet temperature of the heat recovery reservoir is an important parameter in the design of refrigeration with heat recovery systems. In this paper the second law of thermodynamics has been applied to an irreversible Carnot refrigerator with heat recovery (CRHR) coupled to variable-temperature heat reservoirs. The refrigerating rate, input power, refrigeration coefficient, heat recovery coefficient, comprehensive coefficient of performance and exergy efficiency are chosen as the objective functions. The design rule chosen for this study is that the heat transfer area should be constrained. The mathematical expressions for assessing performance parameters with respect to area ratio, were derived for this study. These expressions are transcendental equations. The numerical solution method was employed to calculate the approximate solutions of the optimum performance parameters in a numerical example. The results indicate that the increase in the outlet temperature of heat recovery reservoir could lead to a rise in the maximum value of refrigerating rate and minimum value of input power; also it will lead to the decline in the maximum value of refrigeration coefficient, heat recovery coefficient, comprehensive coefficient and the exergy efficiency. When the ratio of heat recovery heat exchanger area to the summation of high temperature heat exchanger area and the heat recovery heat exchanger area is 1.0, the performance coefficients would attain their limit values and all of the condensing heat could be recycled. Our findings are helpful to the design and optimization to inform preparation of standard relating to the development of refrigerator with heat recovery.

## Introduction

In the last decades, there has been a rapid development to combine space cooling and water heating systems for residences^1^. This combined system is considered to be cost^2^, environmental and energy efficient technology, which recycles some or all of the waste heat in the process of refrigeration for the demand of sanitary water, industrial heating or air reheating and so on^3^. Nowadays, finite-time thermodynamics analysis of thermodynamic systems has become a prominent topic in heat engine, refrigerator and heat pump and so on. Since the 1950s, finite-time thermodynamics has made significant progress after the landmark paper of Novikov^4^ and Chambadal^5^. In the 1970s, Finite-time thermodynamics was further advanced by Curzon and Ahlborn^6^. In recent years, many studies on second law of thermodynamics systems have been presented by many authors^7^. Zhang^8^ et al. studied the optimization of heat exchanger structure based on finite-time thermodynamics. Li Tao^9^ used finite-time thermodynamics to optimize the heat pump system and found that a reasonable selection of the heat transfer area of the heat exchanger can effectively improve the operating performance of the system. John^10^ utilized the Carnot cycle to evaluate the potential for waste heat recovery at a wastewater treatment plants. Chen et al.^11–17^ have analyzed the performances of simple and regenerated, endoreversible and irreversible, constant and variable-temperature heat-reservoir air heat-pumps considering the heat load, heat-load density, coefficient of performance (COP) and so on. Sarkar^18^ studied the minimization of heat exchanger area or overall conductance of heat pumps and refrigerators for a specified capacity and the analytical results were confirmed by a detailed numerical simulation. Wu^19^ proposed an original sinusoidal wavy winglet type vortex generator and evaluated the grade of energy and to explore the irreversible loss during the heat transfer process in view of the second law of thermodynamics. Lei^20^ optimized the heat exchanger area by using the finite time thermodynamics theory. Tan^21^ established an endoreversible Carmot cycle model by using finite time thermodynamics. The surface area distribution of three kinds of heat exchangers was optimized by numerical calculation method, and the maximum output power was obtained. Tyagi et al.^22–25^ applied the finite time thermodynamics to various endoreversible and irreversible cycles. They have investigated the effects of a finite rate of heat transfer or other major irreversibilities on the performance of different cycles. Ruibo^26^ Applying finite-time thermodynamics theory, an irreversible steady flow Lenoir cycle model with variable-temperature heat reservoirs is established, the expressions of power (P) and efficiency (η) are derived. Based on the theory of finite-time thermodynamics, Meng Fankai^27^ designed a channel structure of cooling air, and established a finite-time thermodynamic model of variable temperature heat source thermoelectric cooler based on heat pipe heat dissipation. The thermal resistance of the cold and hot ends of the device was analyzed by numerical simulation method. Wang^28^ built an irreversible Carnot heat engine cycle model for space power plants by using finite time thermodynamics. The influences of internal irreversible effect and heat leakage loss on the optimum power output performance are analyzed, when thermal conductivity coefficients of the heat exchanger and cold exchanger are given. Wu^29^ studied a reversible simple air refrigeration cycle by using classical thermodynamics. Through theoretical analysis and numerical calculations, the optimal performance of the refrigeration cycle is given. The influence of cycle temperature ratio on the optimal performance of refrigeration cycle is analyzed.

The performance of refrigerators with heat recovery coupled to three constant heat reservoirs has been researched using the second law of thermodynamics by our Team^1,5,9,30^. The outlet temperature of the heat recovery reservoir is an important parameter for heat recovery and the variable-temperature heat reservoir is much closer to actual conditions. The performance of the irreversible Carnot refrigerator with heat recovery (CRHR) coupled to variable-temperature heat reservoirs has not been studied by other researchers. El-Din^31^ applied the second law of thermodynamics to irreversible heat pumps and refrigerators with two variable temperature heat reservoirs. *Q*_H_ and *Q*_L_ were chosen to be the objective functions for heat pumps and refrigerators respectively. However, the results are imperfect due to the optimal variable *x* (thermal conductance ratio) contained in *E*_*h*_ and *E*_*c*_; these were treated as an invariable during the process of the derivation for maximization of *Q*_H_ and *Q*_L_. In this paper the second law of thermodynamics was applied to an irreversible Carnot refrigerator with heat recovery coupled to variable-temperature heat reservoirs. The refrigerating rate ($R$), input power ($P$), refrigeration coefficient ($\varepsilon$), heat recovery coefficient ($\varepsilon_{R}$), comprehensive coefficient of performance ($COP_{{\text{int}}}$) and exergy efficiency (${{\varvec{\eta}}}_{\boldsymbol{\Pi }}$) were chosen as the objective functions in this study. Equating the derivatives of those performance parameters with respect to area ratio, *f*, to zero, a group of transcendental equations would be derived. The numerical solution method was employed to calculate the approximate solutions of the optimum performance parameters in a numerical example. The influence of outlet temperature of the heat recovery reservoir on these performance parameters was analyzed in the numerical example.

## Thermodynamic model

**Figure 1:**
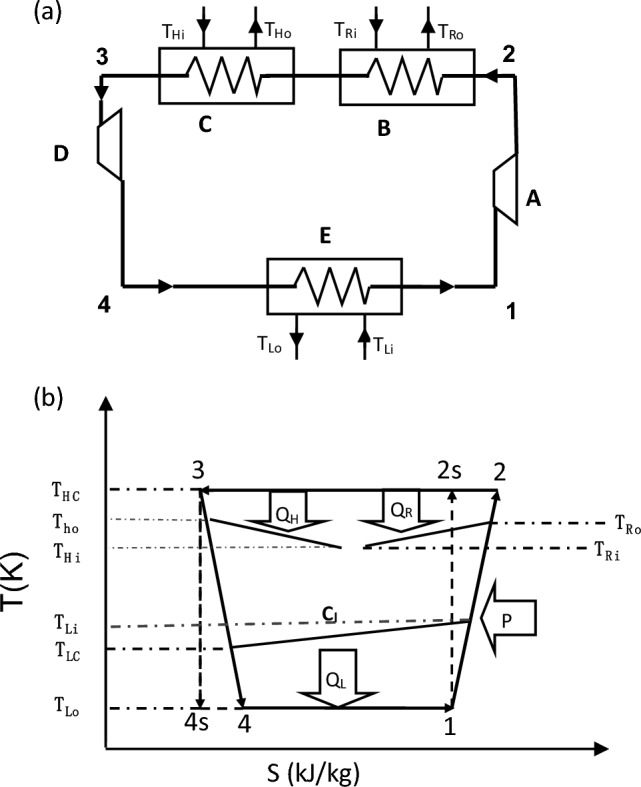
(**a**) The schematic of the refrigerator with heat recovery, (**b**) The temperature–entropy diagram of the CRHR cycle.

An irreversible Carnot refrigerator with heat recovery coupled to variable-temperature heat reservoirs and its surroundings are shown in Fig. 1a. There are three heat exchangers, including high temperature heat exchanger, low temperature heat exchanger and heat recovery heat exchanger, existing in the cycle.

Figure 1b shows the temperature-entropy diagram of an irreversible Carnot refrigerator with heat recovery which operates steadily between three variable temperature reservoirs. The working fluid in the refrigerator has two constant temperatures, $T_{HC}$ and $T_{LC}$. The three heat exchangers are treated as counter flow heat exchanger with finite thermal capacitance rates,$C_{r}$,$C_{h}$ and $C_{l}$.

In Fig. 1, A—the compressor; B—the heat recovery heat exchanger; C—the high temperature heat exchanger; D—the expander, E—the low temperature heat exchanger.

Generally, for a combined space cooling and water heating system, the inlet temperatures of high temperature reservoir ($T_{Hi}$) and heat recovery reservoir ($T_{Ri}$) can be treated as the outdoor environment temperature as described in Eq. (1) and the inlet temperature of low temperature reservoir ($T_{Li}$) can be treated as the indoor environment temperature as given in Eq. (2).

1 $$ T_{Hi} = T_{Ri} = T_{o} $$

2 $$ T_{Li} = T_{i} $$

where $T_{o}$ and $T_{i}$ are the outdoor and indoor environment temperature.

The rate of heat transfer at the high temperature side (Q_H_) is defined by Eq. (3):

3 $$ Q_{H} = C_{h} (T_{Ho} - T_{Hi} ) $$

From Eq. (1), Eq. (3) becomes Eq. (4):

4 $$ Q_{H} = C_{h} (T_{Ho} - T_{o} ) $$

where $C_{h}$ is the thermal capacitance rate of high temperature reservoir and T_Ho_ is the outlet temperature of the high temperature reservoir.

Using the LMTD method, $Q_{H}$ can also be determined by Eq. (5):

5 $$ Q_{H} = U_{h} F_{h} \frac{{(T_{HC} - T_{o} ) - (T_{HC} - T_{Ho} )}}{{\ln \left[ {(T_{HC} - T_{o} )/(T_{HC} - T_{Ho} )} \right]}} $$

where $U_{h}$ is the heat transfer coefficient of high temperature reservoir.$F_{h}$ is the heat transfer area of high temperature reservoir. $T_{HC}$ is the temperature of working fluid at the high temperature side.

From Eqs. (4) and (5), Eq. (6) is derived.

6 $$ \ln \left[ {(T_{HC} - T_{o} )/(T_{HC} - T_{Ho} )} \right] = NTU_{h} $$

From Eqs. (6),  (7) is derived for determination of the outlet temperature of the high temperature reservoir.

7 $$ T_{Ho} = T_{o} + \eta_{h} (T_{HC} - T_{o} ) $$

where $\eta_{h}$ is the effectiveness of the high temperature heat exchanger; and is defined by Eq. (8):

8 $$ \eta_{h} = 1 - \exp ( - NTU_{h} ) $$

Substituting Eq. (7) into Eq. (4) gives Eq. (9):

9 $$ Q_{H} = \eta_{h} C_{h} (T_{HC} - T_{o} ) $$

The rate of heat transfer at the heat recovery side (Q_R_) is determined by Eq. (10):

10 $$ Q_{R} = C_{r} (T_{Ro} - T_{Ri} ) $$

Equation (11) is derived from Eq. (1) and Eq. (10) for determination of Q_R_:

11 $$ Q_{R} = C_{r} (T_{Ro} - T_{o} ) $$

where $C_{r}$ is the thermal capacitance rate of heat recovery reservoir.

Using the LMTD method, $Q_{R}$ can also be determined by Eq. (12), as:

12 $$ Q_{R} = U_{r} F_{r} \frac{{(T_{HC} - T_{o} ) - (T_{HC} - T_{Ro} )}}{{\ln \left[ {(T_{HC} - T_{o} )/(T_{HC} - T_{Ro} )} \right]}} $$

where $U_{r}$ is the heat transfer coefficient of heat recovery reservoir.$F_{r}$ is the heat transfer area of heat recovery reservoir.

From Eqs. (11) and 12), Eq. (13) is derived:

13 $$ \ln \left[ {(T_{HC} - T_{o} )/(T_{HC} - T_{Ro} )} \right] = NTU_{r} $$

From Eqs. (13), (14) is derived:

14 $$ T_{Ro} = T_{o} + \eta_{r} (T_{HC} - T_{o} ) $$

where $\eta_{r}$ is the effectiveness of the heat recovery heat exchanger; and is defined by Eq. (15).

15 $$ \eta_{r} = 1 - \exp ( - NTU_{r} ) $$

Substituting Eq. (14) into Eq. (11) gives Eq. (16):

16 $$ Q_{R} = \eta_{r} C_{r} (T_{HC} - T_{o} ) $$

The rate of heat transfer at the low temperature side, Q_L_ is determined by Eq. (17):

17 $$ Q_{L} = C_{l} (T_{Li} - T_{Lo} ) $$

From Eqs. (2), (18) can be obtained to determine Q_L_:

18 $$ Q_{L} = C_{l} (T_{i} - T_{Lo} ) $$

where $C_{l}$ is the thermal capacitance rate of low temperature reservoir.

Using the LMTD method, $Q_{L}$ can also be determined by Eq. (19), as:

19 $$ Q_{L} = U_{l} F_{l} \frac{{(T_{i} - T_{LC} ) - (T_{Lo} - T_{LC} )}}{{\ln \left[ {(T_{i} - T_{LC} )/(T_{Lo} - T_{LC} )} \right]}} $$

where $U_{l}$ is the heat transfer coefficient of low temperature reservoir.$F_{l}$ is the heat transfer area of low temperature reservoir.

From Eqs. (18) and (19), Eq. (20) is derived:

20 $$ \ln \left[ {(T_{i} - T_{LC} )/(T_{Lo} - T_{LC} )} \right] = NTU_{l} $$

From Eqs. (20), (21) can be derived:

21 $$ T_{Lo} = T_{i} + \eta_{l} (T_{i} - T_{LC} ) $$

where $\eta_{l}$ is the effectiveness of the low temperature heat exchanger; and is defined by Eq. (22).

22 $$ \eta_{l} = 1 - \exp ( - NTU_{l} ) $$

Substituting Eq. (21) into Eq. (18) gives Eq. (23):

23 $$ Q_{L} = \eta_{l} C_{l} (T_{i} - T_{LC} ) $$

The cycle is internally irreversible and the internal irreversibility parameter can be determined by Eq. (24):

24 $$ \frac{{Q_{H} + Q_{R} }}{{T_{HC} }} = \Phi \frac{{Q_{L} }}{{T_{LC}^{{}} }} $$

where $\Phi$ is the internal irreversibility parameter, which is always greater than 1 for an irreversibility cycle and equals to 1 for an endoreversible cycle.

The ratio of the heat transfer rate at the heat recovery side to the total heat emissions, *n* can be defined by Eq. (25), below:

25 $$ n = \frac{{Q_{RC} }}{{Q_{RC} + Q_{HC} }} $$

where, *n* is defined as the heat recovery ratio.

The design rule chosen by this paper is that the heat transfer area should be constrained, as defined by Eq. (26):

26 $$ F_{h} + F_{r} + F_{l} = F $$

The ratio of *F*_*h*_ and *F*_*r*_ to *F* is defined by Eq. (27):

27 $$ f = \frac{{F_{h} + F_{r} }}{F} $$

And Eq. (28) defines the ratio of *F*_*r*_ to *F*_*h*_ and *F*_*r*_.

28 $$ f_{0} = \frac{{F_{r} }}{{F_{h} + F_{r} }} $$

where $f$ is the ratio of the summation of high temperature heat exchanger area and the heat recovery heat exchanger area to the total heat exchanger area. $f_{0}$ is the ratio of heat recovery heat exchanger area to the summation of high temperature heat exchanger area and the heat recovery heat exchanger area.

From Eqs. (26), (27) and Eqs. (28), (29), (30) and (31) are respectively derived:

29 $$ F_{h} = f(1 - f_{0} )F $$

30 $$ F_{r} = ff_{0} F $$

31 $$ F_{l} = (1 - f)F $$

The equations above constitute the mathematical model for optimizing the performance of an irreversible Carnot refrigerator with heat recovery operating between the three variable-temperature heat reservoirs. From these equations, Eqs. (32) to (36) can be derived for description of the objective functions as given below:

32 $$ R = Q_{L} = C_{l} \eta_{l} T_{i} - C_{l} \eta_{l} T_{i} \left[ {\frac{{(T_{Ro} - T_{o} )(C_{h} \eta_{h} + C_{r} \eta_{r} )}}{{\Phi C_{l} \eta_{l} (T_{Ro} - T_{o} + \eta_{r} T_{o} )}} + 1} \right]^{ - 1} $$

33 $$ P = Q_{H} + Q_{R} - Q_{L} = [(C_{h} \eta_{h} + C_{r} \eta_{r} )\frac{{T_{Ro} - T_{o} }}{{\eta_{r} }} - C_{l} \eta_{l} T_{i} + C_{l} \eta_{l} T_{i} [\frac{{(T_{Ro} - T_{o} )(C_{h} \eta_{h} + C_{r} \eta_{r} )}}{{\Phi C_{l} \eta_{l} (T_{Ro} - T_{o} + \eta_{r} T_{o} )}} + 1]^{ - 1} $$

34 $$ \varepsilon = \frac{{Q_{L} }}{{Q_{H} + Q_{R} - Q_{L} }} = \frac{{C_{l} \eta_{l} T_{i} - C_{l} \eta_{l} T_{i} [\frac{{(T_{Ro} - T_{o} )(C_{h} \eta_{h} + C_{r} \eta_{r} )}}{{\Phi C_{l} \eta_{l} (T_{Ro} - T_{o} + \eta_{r} T_{o} )}} + 1]^{ - 1} }}{{[(C_{h} \eta_{h} + C_{r} \eta_{r} )\frac{{T_{Ro} - T_{o} }}{{\eta_{r} }}C_{l} \eta_{l} T_{i} - C_{l} \eta_{l} T_{i} [\frac{{(T_{Ro} - T_{o} )(C_{h} \eta_{h} + C_{r} \eta_{r} )}}{{\Phi C_{l} \eta_{l} (T_{Ro} - T_{o} + \eta_{r} T_{o} )}} + 1]^{ - 1} }} $$

35 $$ \varepsilon_{R} = \frac{{Q_{R} }}{{Q_{H} + Q_{R} - Q_{L} }} = \frac{{(T_{Ro} - T_{o} )C_{r} }}{{[(C_{h} \eta_{h} + C_{r} \eta_{r} )\frac{{T_{Ro} - T_{o} }}{{\eta_{r} }}C_{l} \eta_{l} T_{i} - C_{l} \eta_{l} T_{i} [\frac{{(T_{Ro} - T_{o} )(C_{h} \eta_{h} + C_{r} \eta_{r} )}}{{\Phi C_{l} \eta_{l} (T_{Ro} - T_{o} + \eta_{r} T_{o} )}} + 1]^{ - 1} }} $$

36 $$ COP_{{\text{int}}} = \varepsilon + \varepsilon_{R} = \frac{{C_{l} \eta_{l} T_{i} - C_{l} \eta_{l} T_{i} [\frac{{(T_{Ro} - T_{o} )(C_{h} \eta_{h} + C_{r} \eta_{r} )}}{{\Phi C_{l} \eta_{l} (T_{Ro} - T_{o} + \eta_{r} T_{o} )}} + 1]^{ - 1} + (T_{Ro} - T_{o} )C_{r} }}{{[(C_{h} \eta_{h} + C_{r} \eta_{r} )\frac{{T_{Ro} - T_{o} }}{{\eta_{r} }}C_{l} \eta_{l} T_{i} - C_{l} \eta_{l} T_{i} [\frac{{(T_{Ro} - T_{o} )(C_{h} \eta_{h} + C_{r} \eta_{r} )}}{{\Phi C_{l} \eta_{l} (T_{Ro} - T_{o} + \eta_{r} T_{o} )}} + 1]^{ - 1} }} $$

37 $$ \eta_{\Pi } = \frac{{E_{L} + E_{R} }}{{E_{p} }} = \left[ {\frac{{2T_{o} + \frac{{\eta_{h} \left( {T_{Ro} - T_{o} } \right)}}{{\eta_{r} }}}}{{2T_{i} - \frac{P}{{C_{l} }}}} - 1} \right]\varepsilon + \left[ {1 - \frac{{2T_{o} + \frac{{\eta_{h} \left( {T_{Ro} - T_{o} } \right)}}{{\eta_{r} }}}}{{T_{Ro} + T_{o} }}} \right]\varepsilon_{R} $$

In these equations, $\eta_{h} = 1 - \exp [ - U_{h} (1 - f_{0} )\dot{f}_{{}} F/C_{h} ]$, $\eta_{r} = 1 - \exp [ - U_{r} \dot{f}f_{0} F/C_{{\text{r}}} ]$, $\eta_{l} = 1 - \exp [ - U_{l} (1 - \dot{f})F/C_{l} ]$.

Where $R$ is the refrigeration rate; $P$ is the input power; $\varepsilon$ is the refrigeration coefficient; $\varepsilon_{R}$ is the heat recovery coefficient; which is defined as the ratio of heat recovery rate to the input power. $COP_{{\text{int}}}$ is the comprehensive coefficient, which is the summation of refrigeration coefficient and heat recovery coefficient. $E_{p}$ is the input electricity exergy of the CRHR; $E_{L}$ is the cold exergy; $E_{R}$ is the recovery heat exergy; $E_{H}$ is the heat exergy emitted to the surroundings directly, which is a part of the exergy loss; $\Pi$ is the other exergy loss of the CRHR.

In Eqs. (32) to (37) the superscript point on $f$ means that $f$ is chosen to be the optimization variable when the parameters such as $C_{h}$,$C_{r}$,$C_{l}$,$U_{h}$,$U_{r}$,$U_{l}$,$F$,$T_{i}$,$T_{o}$,$T_{Ro}$,$f_{o}$ and $\Phi$ are specified. Maximizations or minimizations of these performance parameters with respect to $f$ give Eqs. (38) to (43):

38 $$ dR/df \ge 0 $$

39 $$ dP/df \ge 0 $$

40 $$ d\varepsilon /df \ge 0 $$

41 $$ d\varepsilon_{R} /df \ge 0 $$

42 $$ dCOP_{{\text{int}}} /df \ge 0 $$

43 $$ d\eta_{\Pi } /df \ge 0 $$

By calculating $dR/df = 0$,$dP/df = 0$,$d\varepsilon /df = 0$,$d\varepsilon_{R} /df = 0$_,_$dCOP_{{\text{int}}} /df = 0$ and $d\eta_{\Pi } /df \ge 0$; the maximums/minimums of $R$,$P$,$\varepsilon$,$\varepsilon_{R}$, ${\text{COP}}_{{{\text{int}}}}$ and $\eta_{\Pi }$ can be obtained. From Eqs. (32) to (43), six of the objective functions are obviously the transcendental equations and the analytical formulas cannot be obtained for the optimum performance parameters. In this paper, the numerical solution method was used to calculate the approximate solution by Matlab software.The influence of temperature variations of heat recovery reservoir on the optimal performance parameters can be researched by substituting different values of $T_{Ro}$. In this way, the influence of $f_{0}$ and $\Phi$ also can be studied.

## Results and discussion

The specified parameters and numerical examples are listed in Table 1^30,31^:

**Table 1:** Specified parameters.

$$C_{h}$$ $${\text{kW}}/{\text{K}}$$	$$C_{r}$$$${\text{kW}}/{\text{K}}$$	$$C_{l}$$$${\text{kW}}/{\text{K}}$$	$$F$$ $${\text{m}}^{2}$$	$$U_{h}$$$${\text{kW}}/\left( {{\text{K}}\;{\text{m}}^{2} } \right)$$	$$U_{r}$$$${\text{kW}}/\left( {{\text{K}}\;{\text{m}}^{2} } \right)$$	$$U_{l}$$$${\text{kW}}/\left( {{\text{K}}\;{\text{m}}^{2} } \right)$$	$$T_{i}$$$${\text{K}}$$	$$T_{o}$$$${\text{K}}$$
5.0	3.0	5.0	4	1.0	1.0	1.0	293	308

There are two main input variables in this paper, one of which is that the value range of the ratio of the summation of high temperature heat exchanger area and the heat recovery heat exchanger area to the total heat exchanger area ($f_{{}}$) is 0–1, and the other is $T_{Ro}$, which is the outlet temperature of heat recovery reservoir. Because the heat recovery in this paper is used to produce sanitary water and industrial heating. Therefore, the lowest temperature of the $T_{Ro}$ must be higher than the ambient temperature, and the highest temperature should not exceed the temperature of the condenser in the refrigeration cycle. Hence, in this paper, the temperature range of the $T_{Ro}$ is selected as 310-370 K.

### Optimal value of $R$

**Figure 2:**
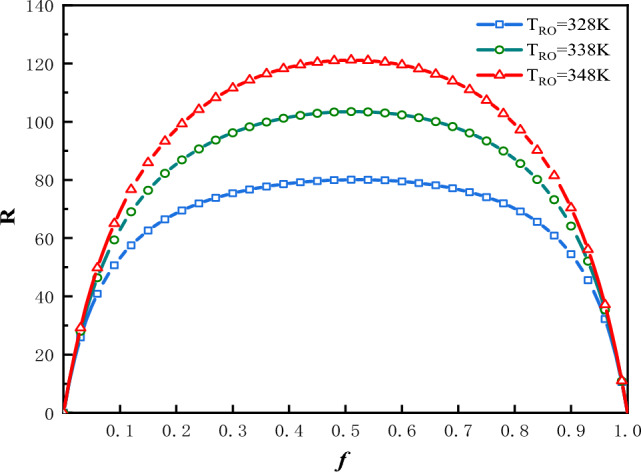
$R$ vs. $f$ for three values of $T_{Ro}$.

**Figure 3:**
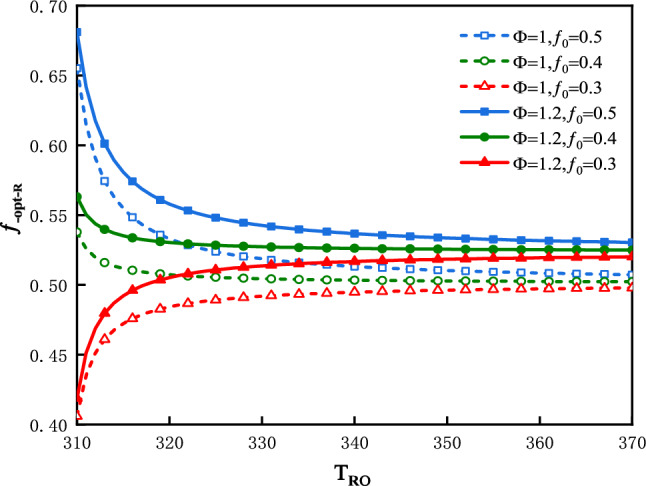
$f_{ - opt - R}$ vs. $T_{Ro}$ for three values of $f_{0}$ and two values of $\Phi$.

The plot of $R$ vs. $f$ is shown in Fig. 2 for three values of $T_{Ro}$ under the condition that: $\Phi$ = 1 and $f_{0}$ = 0.5. The plot of $f_{ - opt - R}$ vs.$T_{Ro}$ is shown in Fig. 3 for three values of $f_{0}$ and two values of $\Phi$.

In Fig. 2, the optimal values of $f$ denoted as $f_{ - opt - R}$, at which the refrigerating rate would attain their maximum values, are denoted as $R_{f}$. In this case the optimal values of $f$ are $f_{ - opt - R}$ = 0.508, 0.505 and 0.503; the maximums of refrigeration rate are $R_{f}$ = 80.07 kW, 103.45 kW and 121.18 kW corresponding to $T_{Ro}$ = 328 K, 338 K and 348 K respectively. The greater is the $T_{Ro}$, the greater would be the refrigeration rate. Figure 3 shows that $T_{Ro}$**,**$\Phi$ and $f_{0}$ all have effects on $f_{ - opt - R}$. The value of $f_{ - opt - R}$ moves closer to a constant value gradually, along with an increase in $T_{Ro}$. This constant value is influenced by $\Phi$. The smaller is the value of $f_{0}$, the smaller would be the $f_{ - opt - R}$.

**Figure 4:**
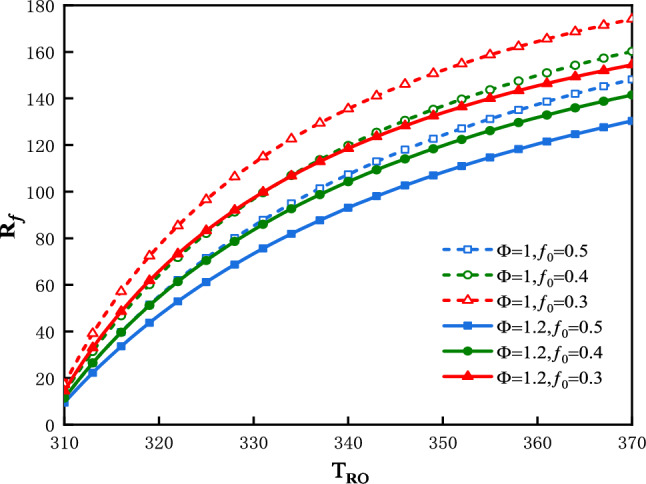
$R_{f}$ vs. $T_{Ro}$ for three values of $f_{0}$ and two values of $\Phi$.

The plot of $R_{f}$ vs.$T_{Ro}$ is shown in Fig. 4 for three values of $f_{0}$ and two values of $\Phi$. Figure 4 shows an increase in $R_{f}$ with an almost corresponding increase in $T_{Ro}$. The smaller is the value of $f_{0}$, the greater would be the $R_{f}$**.** The greater is the value of $\Phi$, the smaller would be $R_{f}$**.**

### Optimal value of $P$

**Figure 5:**
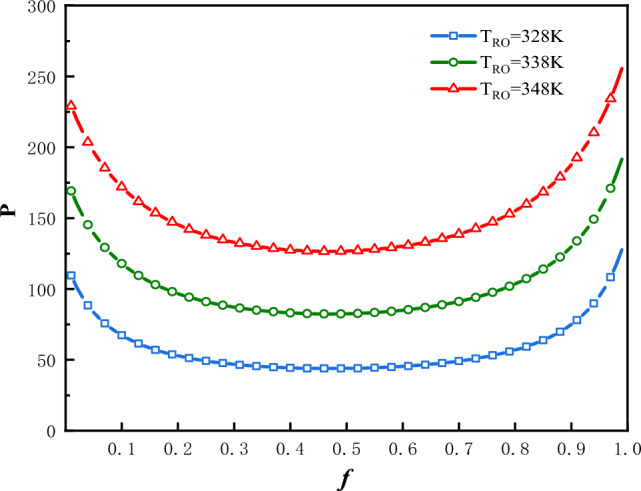
$P$ vs.$f$ for three values of $T_{Ro}$.

The plot of $P$ vs.$f$ is shown in Fig. 5 for three values of $T_{Ro}$ under the condition that:$\Phi$ = 1 and $f_{0}$ = 0.5. In Fig. 5, the optimal values of $f$ denoted as $f_{ - opt - P}$, at which the input power attain minimum values are denoted as $P_{f}$. In this case the optimal values of $f$ are $f_{ - opt - P}$ = 0.467, 0.465 and 0.463. The minimum values of input power are $P_{f}$** = **43.86 kW, 82.38 kW and 126.58 kW, corresponding to $T_{Ro}$** = **328 K, 338 K and 348 K respectively. The greater is the $T_{Ro}$, the greater would be the input power.

**Figure 6:**
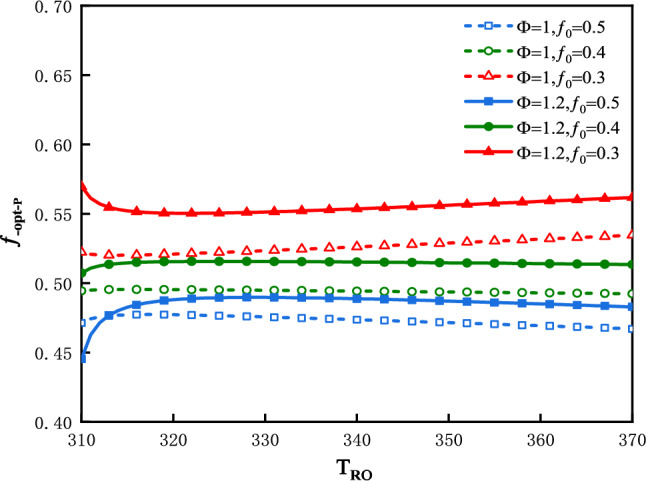
$f_{ - opt - P}$ vs. $T_{Ro}$ for three values of $f_{0}$ and two values of $\Phi$.

The plot of $f_{ - opt - P}$ vs. $T_{Ro}$ is shown in Fig. 6 for three values of $f_{0}$ and two values of $\Phi$. Figure 6 shows that $T_{Ro}$**,** $\Phi$ and $f_{0}$ all have effects on $f_{ - opt - P}$. Different from the curves of $f_{ - opt - R}$ vs.$T_{Ro}$**,** the curves of $f_{ - opt - P}$ diverge from each other along with an increase in $T_{Ro}$.

**Figure 7:**
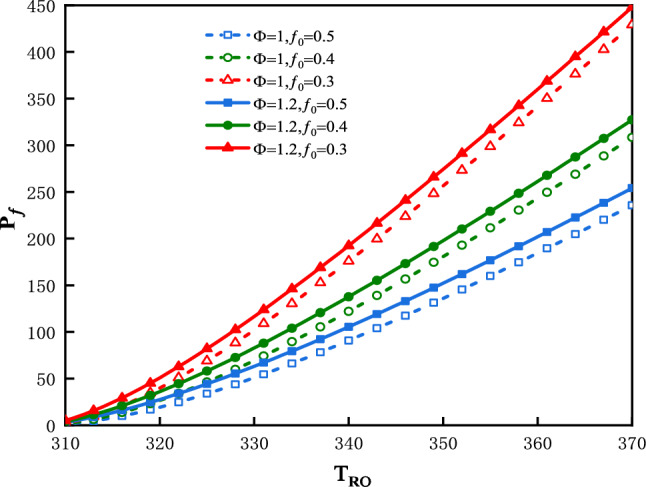
$P_{f}$ vs. $T_{Ro}$ for three values of $f_{0}$ and two values of $\Phi$.

The plot of $P_{f}$ vs. $T_{Ro}$ is shown in Fig. 7 for three values of $f_{0}$ and two values of $\Phi$. Figure 7 shows that $P_{f}$ increase along with an increase in $T_{Ro}$**.** The smaller is the value of $f_{0}$, the greater would be the $P_{f}$**.** The greater is the value of $\Phi$, the greater is $P_{f}$**.**

### Optimal value of $\varepsilon$

**Figure 8:**
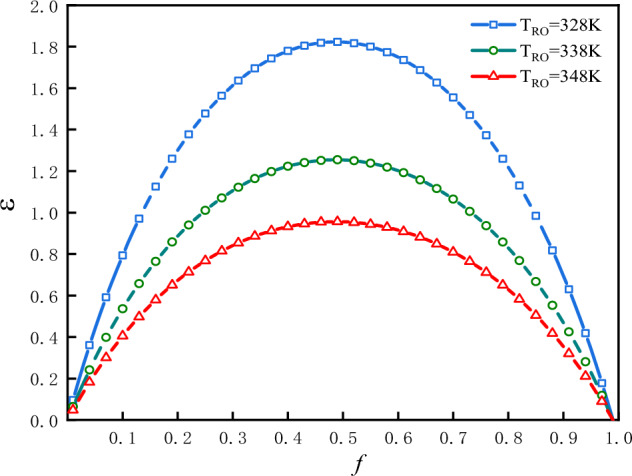
$\varepsilon$ vs. $f$ for three values of $T_{Ro}$.

The plot of $\varepsilon$ vs.$f$ is shown in Fig. 8 for three values of $T_{Ro}$ under the condition that: $\Phi$ = 1 and $f_{0}$ = 0.5. In Fig. 8, there is only one optimal value of $f$, denoted as $f_{ - opt - \varepsilon }$, at which the refrigeration coefficient attains maximum values, denoted as $\varepsilon_{f}$. In this case the optimal value of $f$ is $f_{ - opt - \varepsilon }$ = 0.483; the maximum values of refrigeration coefficient are $\varepsilon_{f}$** = **1.83, 1.26 and 0.96 corresponding to $T_{Ro}$** = **328 K, 338 K and 348 K respectively. The greater is the $T_{Ro}$, the smaller would be the refrigeration coefficient.

**Figure 9:**
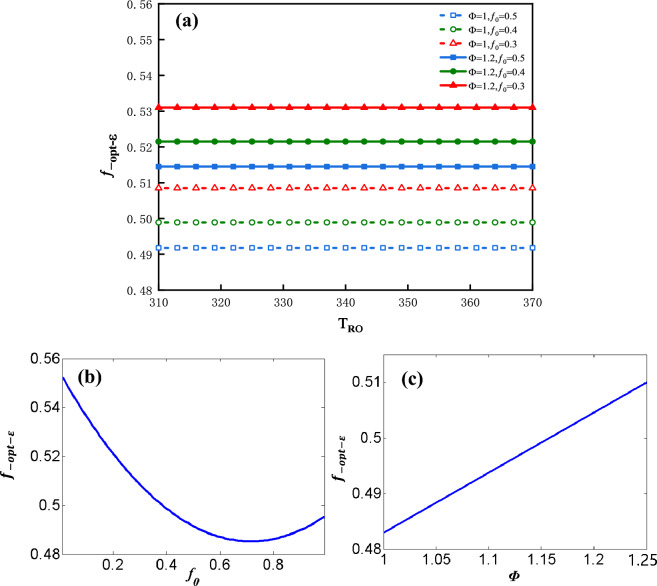
(**a**) $f_{ - opt - \varepsilon }$ vs. $T_{Ro}$ for three values of $f_{0}$ and two values of $\Phi$, (**b**) $f_{ - opt - \varepsilon }$ vs. $f_{0}$, (**c**) $f_{ - opt - \varepsilon }$ vs. $\Phi$.

The influence factors on $f_{ - opt - \varepsilon }$ can be analyzed by the following plots. Figure 9a shows the plot of $f_{ - opt - \varepsilon }$ vs. $T_{Ro}$ for three values of $f_{0}$ and two values of $\Phi$. $T_{Ro}$ is shown to have no effect on $f_{ - opt - \varepsilon }$. Figure 9b shows the plot of $f_{ - opt - \varepsilon }$ vs. $f_{0}$ under the condition that: $\Phi$ = 1 and $T_{Ro}$ = 328* K*. In the range of the available value of $f_{0}$,$0 \le f_{0} \le 1$, $f_{ - opt - \varepsilon }$ has a maximum value and a minimum value. When $f_{0}$ = 0, $f_{ - opt - \varepsilon }$ attains its maximum value (about 0.55); when $f_{0}$ = 0.7, $f_{ - opt - \varepsilon }$ attains its minimum value (about 0.481). Figure 9c shows the plot of $f_{ - opt - \varepsilon }$ vs. $\Phi$ under the condition that $f_{0}$ = 0.5 and $T_{Ro}$ = 328* K*. $f_{ - opt - \varepsilon }$ vs. $\Phi$ is approximate to the monotonous linear relationship. The greater is the $\Phi$, the greater would be the value of $f_{ - opt - \varepsilon }$.

**Figure 10:**
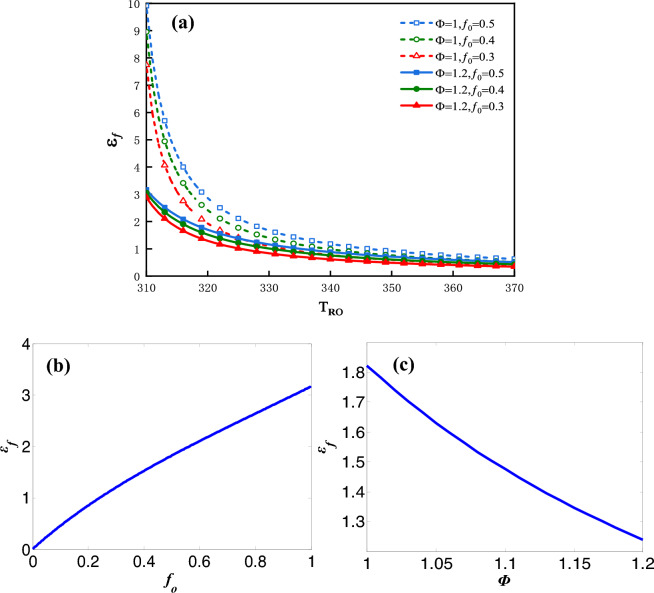
(**a**) $\varepsilon_{f}$ vs. $T_{Ro}$ for three values of $f_{0}$ and two values of $\Phi$, (**b**) $\varepsilon_{f}$ vs. $f_{0}$, (**c**) $\varepsilon_{f}$ vs. $\Phi$.

The influence factors on $\varepsilon_{f}$ can be analyzed by the following plots. Figure 10a shows the plot of $\varepsilon_{f}$ vs. $T_{Ro}$ for three values of $f_{0}$ and two values of $\Phi$.

The maximum $\varepsilon_{f}$ decreases dramatically along with the increase of $T_{Ro}$. From Eq. (11) the heat recovery rate $Q_{R}$ is shown to increase monotonically with $T_{Ro}$ due to the specified values of $T_{o}$ and $C_{r}$. That means the greater is $T_{Ro}$, the larger would be the recycled heat and the lower is refrigeration coefficient. Figure 10b shows the plot of $\varepsilon_{f}$ vs.$f_{0}$ under the condition that $\Phi$ = 1 and $T_{Ro}$ = 328* K* and Fig. 10c shows the plot of $\varepsilon_{f}$ vs.$\Phi$ under the condition that $f_{0}$ = 0.5 and $T_{Ro}$ = 328* K*. $\varepsilon_{f}$ vs.$f_{0}$ as well as $\varepsilon_{f}$ vs. $\Phi$ are approximate to the monotonous linear relationships. The greater is the $f_{0}$, the greater would be the value of $\varepsilon_{f}$. The greater is the $\Phi$, the smaller would be $\varepsilon_{f}$.

### Optimal value of $\varepsilon_{R}$

**Figure 11:**
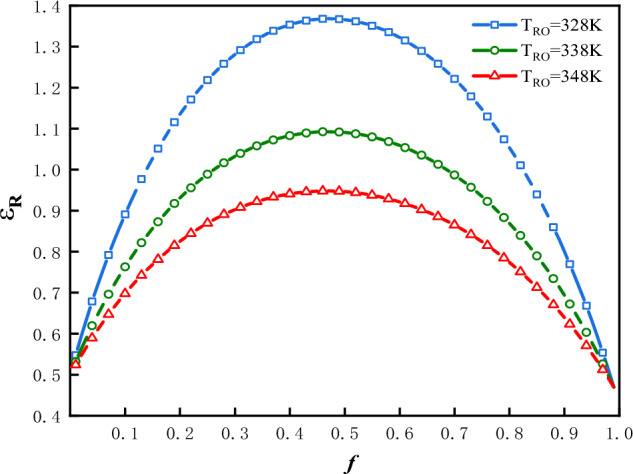
$\varepsilon_{R}$ vs. $f$ for three values of $T_{Ro}$.

The plot of $\varepsilon_{R}$ vs.$f$ is shown in Fig. 11 for three values of $T_{Ro}$ under the condition that: $\Phi$ = 1 and $f_{0}$ = 0.5. In Fig. 11, the optimal value of $f$ is denoted as $f_{ - opt - \varepsilon R}$, at which the heat recovery coefficient attains its maximum values, denoted as $\varepsilon_{Rf}$. In this case the optimal values of $f$ are $f_{ - opt - \varepsilon R}$ = 0.467, 0.465 and 0.463. The maximum values of refrigeration coefficient are $\varepsilon_{f}$ = 1.37, 1.09 and 0.95 corresponding to $T_{Ro}$ = 328 K, 338 K and 348 K respectively. The values of $f_{ - opt - \varepsilon R}$ is the same as $f_{ - opt - P}$ due to the definition of $\varepsilon_{R}$ in Eq. (35). The greater is the $T_{Ro}$, the smaller would be the heat recovery coefficient.

**Figure 12:**
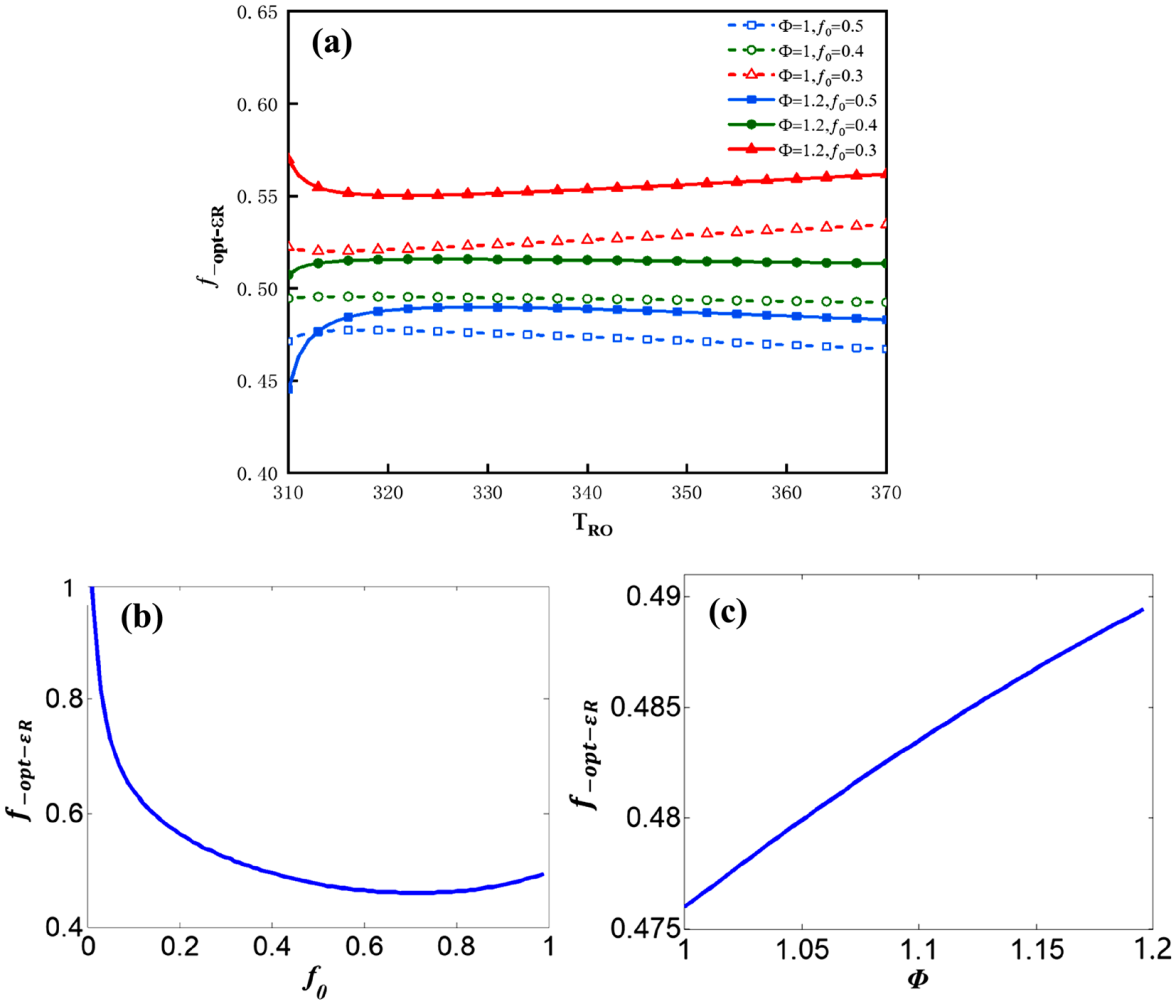
(**a**) $f_{ - opt - \varepsilon R}$ vs. $T_{Ro}$ for three values of $f_{0}$ and two values of $\Phi$, (**b**) $f_{ - opt - \varepsilon R}$ vs $f_{0}$, (**c**) $f_{ - opt - \varepsilon R}$ vs. $\Phi$.

The influence factors on $f_{ - opt - \varepsilon R}$ can be analyzed by the following plots. Figure 12a shows the plot of $f_{ - opt - \varepsilon R}$ vs.$T_{Ro}$ for three values of $f_{0}$ and two values of $\Phi$. The curves in Fig. 12a are the same as Fig. 6. Figure 12b shows the plot of $f_{ - opt - \varepsilon R}$ vs.$f_{0}$ under the condition that $\Phi$ = 1 and $T_{Ro}$ = 328* K*. In the range of the available value of $f_{0}$, ($0 \le f_{0} \le 1$)$f_{\varepsilon R}$ has a minimum value. When $f_{0}$ = 0, $f_{ - opt - \varepsilon R}$ gradually tend to be 1; when $f_{0}$ = 0.72, $f_{ - opt - \varepsilon R}$ attains its minimum value (about 0.46). Figure 12c shows the plot of $f_{{\varepsilon_{R} }}$ vs.$\Phi$ under the condition that $f_{0}$ = 0.5 and $T_{Ro}$ = 328* K*. $f_{ - opt - \varepsilon }$ vs.$\Phi$ is approximate to the monotonous linear relationship. The greater is the $\Phi$, the greater would be the value of $f_{ - opt - \varepsilon R}$.

**Figure 13:**
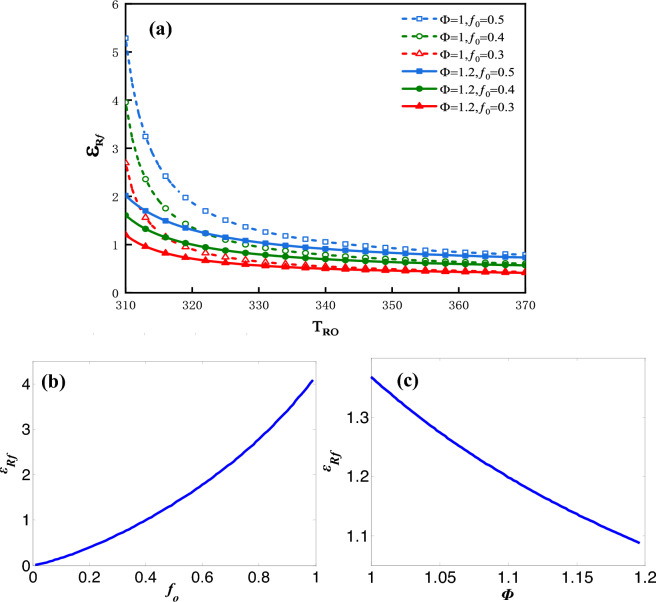
(**a**) $\varepsilon_{Rf}$ vs. $T_{Ro}$ for three values of $f_{0}$ and two values of $\Phi$, (**b**) $\varepsilon_{Rf}$ vs. $f_{0}$, (**c**) $\varepsilon_{Rf}$ vs. $\Phi$.

The influence factors on $\varepsilon_{Rf}$ can be analyzed by the following plots. Figure 13a shows the plot of $\varepsilon_{Rf}$ vs. $T_{Ro}$ for three values of $f_{0}$ and two values of $\Phi$. The maximum $\varepsilon_{Rf}$ declines sharply along with an increase in $T_{Ro}$, due to the input power $P$ is increasing faster than $Q_{R}$. Figure 13b shows the plot of $\varepsilon_{Rf}$ vs.$f_{0}$ under the condition that $\Phi$ = 1 and $T_{Ro}$ = 328* K* and Fig. 13c shows the plot of $\varepsilon_{Rf}$ vs.$\Phi$ under the condition that $f_{0}$ = 0.5 and $T_{Ro}$ = 328* K*. $\varepsilon_{f}$ vs.$f_{0}$ as well as $\varepsilon_{f}$ vs. $\Phi$ are approximate to the monotonous linear relationships. The influence rules of $f_{0}$ and $\Phi$ on $\varepsilon_{Rf}$ is similar to $\varepsilon_{f}$.

### Optimal value of $COP_{{\text{int}}}$

From definition of $COP_{{\text{int}}}$ in Eq. (36) that there must be an optimal value of $f$, denoted as $f_{{ - opt - COP{\text{int}} }}$, at which the comprehensive coefficient could attain its maximum values, denoted as $COP_{{{\text{int}} f}}$.

**Figure 14:**
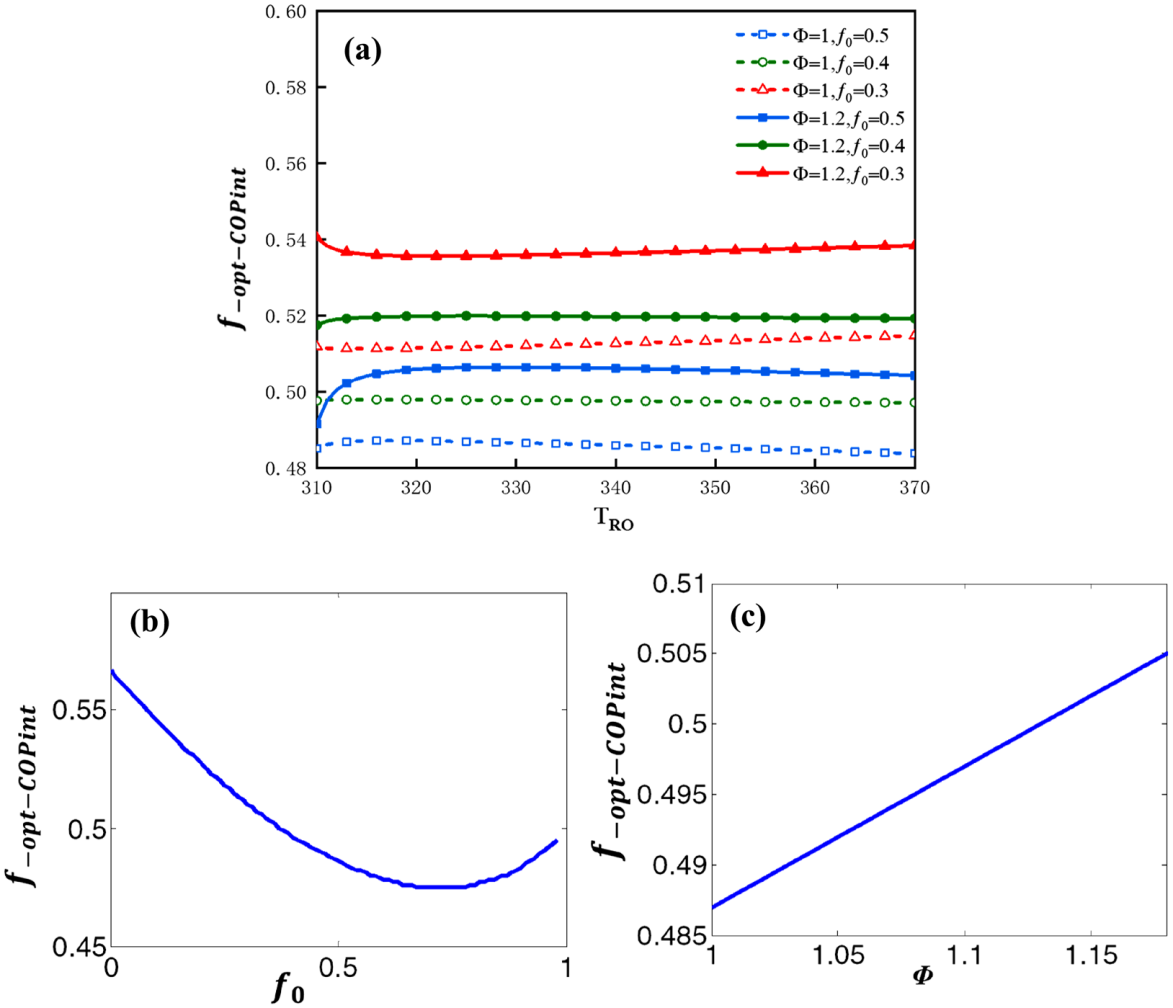
(**a**) $f_{{ - opt - COP{\text{int}} }}$ vs. $T_{Ro}$ for three values of $f_{0}$ and two values of $\Phi$, (**b**) $f_{{ - opt - COP{\text{int}} }}$ vs. $f_{0}$, (**c**) $f_{{ - opt - COP{\text{int}} }}$ vs. $\Phi$.

The influence factors on $\varepsilon_{Rf}$ can be analyzed by the following plots. Figure 14a shows the plot of $f_{{ - opt - COP{\text{int}} }}$ vs. $T_{Ro}$ for three values of $f_{0}$ and two values of $\Phi$. The curves in Fig. 14a are almost horizontal straight lines. The variation of $T_{Ro}$ has a small influence on $f_{{ - opt - COP{\text{int}} }}$. Figure 14b shows the plot of $f_{{ - opt - COP{\text{int}} }}$ vs. $f_{0}$ under the condition that $\Phi$ = 1 and $T_{Ro}$ = 328* K*. In the range of the available value of $f_{0}$, ($0 \le f_{0} \le 1$) $f_{ - opt - \varepsilon R}$ has a maximum value and a minimum value. When $f_{0}$ = 0, $f_{ - opt - \varepsilon }$ attains its maximum value (0.56); when $f_{0}$ = 0.71, $f_{ - opt - \varepsilon }$ attains its minimum value (about 0.473). Figure 14c shows the plot of $f_{{ - opt - COP{\text{int}} }}$ vs. $\Phi$ under the condition that $f_{0}$ = 0.5 and $T_{Ro}$ = 328* K*. $f_{{ - opt - COP{\text{int}} }}$ vs. $\Phi$ is approximate to the monotonous linear relationship. The greater is the $\Phi$, the greater would be the value of $f_{{ - opt - COP{\text{int}} }}$.

**Figure 15:**
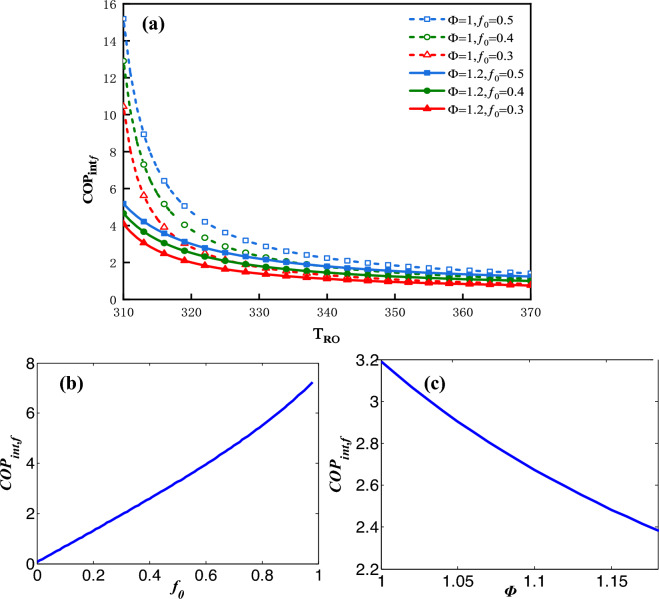
(**a**) $COP_{{{\text{int}} f}}$ vs. $T_{Ro}$ for three values of $f_{0}$ and two values of $\Phi$, (**b**) $COP_{{{\text{int}} f}}$ vs. $f_{0}$, (c) $COP_{{{\text{int}} f}}$ vs. $\Phi$.

The influence factors on $COP_{{{\text{int}} f}}$ can be analyzed by the following plots. Figure 15a shows the plot of $COP_{{{\text{int}} f}}$ vs. $T_{Ro}$ for three values of $f_{0}$ and two values of $\Phi$. The maximum $COP_{{{\text{int}} f}}$ declines sharply along with an increase in $T_{Ro}$. Figure 15b shows the plot of $COP_{{{\text{int}} f}}$ vs.$f_{0}$ under the condition that $\Phi$ = 1 and $T_{Ro}$ = 328* K* and Fig. 15c shows the plot of $COP_{{{\text{int}} f}}$ vs. $\Phi$ under the condition that $f_{0}$ = 0.5 and $T_{Ro}$ = 328* K*. $COP_{{{\text{int}} f}}$ vs. $f_{0}$ as well as $COP_{{{\text{int}} f}}$ vs.$\Phi$ are approximate to the monotonous linear relationships.

Figures 2, 3, 4, 5, 6, 7, 8, 9, 10, 11, 12, 13, 14, and 15 show the completed approximate solutions of a numerical example. The results of this numerical solution example are under the specified conditions, but the influence factors on the performance parameters are of general significance. With specified heat transfer coefficients and thermal capacitance rates there must be a maximum value of the refrigerating rate, refrigeration coefficient, heat recovery coefficient or comprehensive coefficient and a minimum value of the input power existing in the cycle. The increase of the outlet temperature of heat recovery reservoir could lead to a rise in the maximum value of refrigeration rate and minimum value of input power as well as to the decline of the maximum value of refrigeration coefficient, heat recovery coefficient and comprehensive coefficient. The rise of $f_{0}$ is beneficial to the performance coefficients. The rise of $\Phi$ is harmful to them.

### Optimal value of $\eta_{\Pi }$

**Figure 16:**
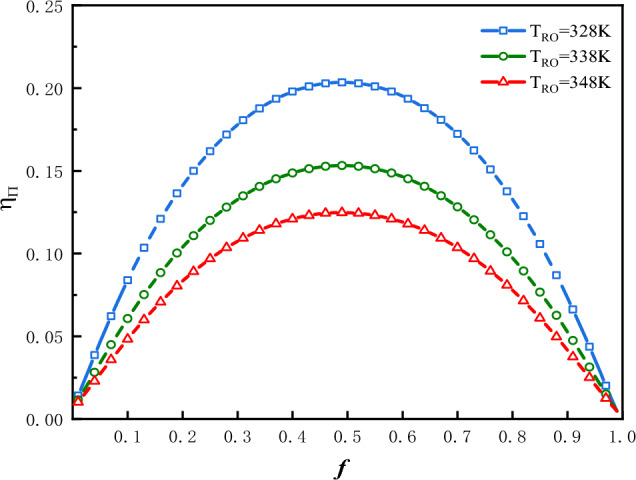
$\eta_{\Pi }$ vs. $f$ for three values of $T_{Ro}$.

**Figure 17:**
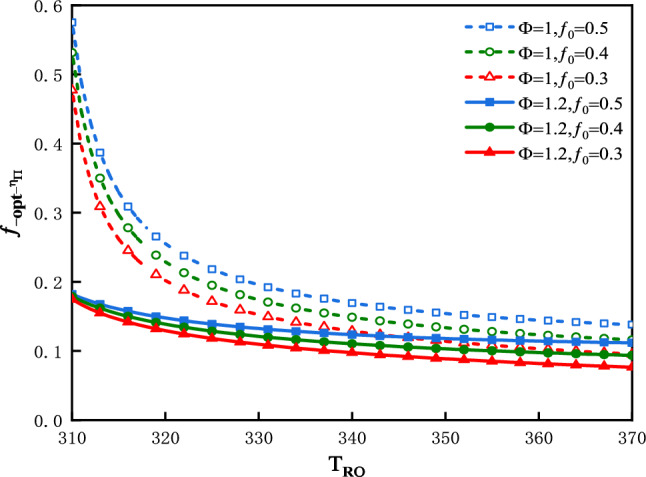
$f_{{ - opt - \eta_{\Pi } }}$ vs. $T_{Ro}$ for three values of $f_{0}$ and two values of $\Phi$.

The plot of $\eta_{\Pi }$ vs. $f$ is shown in Fig. 16 for three values of $T_{Ro}$ under the condition that: $\Phi$ = 1 and $f_{0}$ = 0.5. The plot of $f_{{ - opt - \eta_{\Pi } }}$ vs.$T_{Ro}$ is shown in Fig. 17 for three values of $f_{0}$ and two values of $\Phi$.

In Fig. 16, the optimal values of $\eta_{\Pi }$ denoted as $f_{{ - opt - \eta_{\Pi } }}$, at which the exergy efficiency would attain their maximum values, are denoted as $\eta_{{\Pi_{f} }}$. In this case the optimal values of $\eta_{\Pi }$ are $f_{{ - opt - \eta_{\Pi } }}$ = 0.4942, 0.4958 and 0.4927; the maximums of exergy efficiency are $\eta_{{\Pi_{f} }}$ = 0.2035, 0.1532 and 0.1248 corresponding to $T_{Ro}$ = 328 K, 338 K and 348 K respectively. The greater is the $T_{Ro}$, the smaller would be the exergy efficiency. Figure 17 shows that $T_{Ro}$,$\Phi$ and $f_{0}$ all have effects on $f_{{ - opt - \eta_{\Pi } }}$. The value of $f_{{ - opt - \eta_{\Pi } }}$ moves closer to a constant value gradually, along with an increase in $T_{Ro}$. This constant value is influenced by $\Phi$. The smaller is the value of $f_{0}$, the smaller would be the $f_{{ - opt - \eta_{\Pi } }}$.

## Conclusion

In this paper, we applied the second law of thermodynamics to an irreversible Carnot refrigerator with heat recovery coupled to variable-temperature heat reservoirs. The heat recovery process involves the recycling of waste heat generated during refrigeration for various purposes, such as sanitary water supply, industrial heating, and air condensation. Through the use of numerical solutions, we were able to obtain accurate results for a specific example. Our study yielded several key findings, which are summarized below:

1. With specified heat transfer coefficients and thermal capacitance rates there must be an optimal value of $f$ at which the performance parameter attains maximum or minimum value. The outlet temperature of heat recovery reservoir ($T_{Ro}$) can have an effect on $f_{ - opt - R}$,$f_{ - opt - P}$,$f_{{ - opt - \varepsilon_{R} }}$,$f_{{ - opt - COP_{{\text{int}}} }}$ and $f_{{ - opt - \eta_{\Pi } }}$; but it has no effect on $f_{ - opt - \varepsilon }$.
2. The increase in the outlet temperature of heat recovery reservoir ($T_{Ro}$) could lead to a rise in the maximum value of refrigerating rate ($R_{f}$) and minimum value of input power ($P_{f}$); also it will lead to the decline in the maximum value of refrigeration coefficient ($\varepsilon_{f}$), heat recovery coefficient ($\varepsilon_{Rf}$), comprehensive coefficient ($COP_{{{\text{int}} f}}$) and the exergy efficiency ($\eta_{\Pi f}$).
3. The rise of $f_{0}$ is beneficial to the performance coefficients, but it could lead to a decline in $R_{f}$. When $f_{0}$ = 1.0 the performance coefficients would attain their limit values and all of the condensing heat could be recycled. The rise of $\Phi$ can be harmful to the performance coefficients.

## Data Availability

The datasets used and/or analysed during the current study available from the corresponding author on reasonable request.

## List of symbols

$C$: Thermal capacitance rate (kW K^−^^1^)
$$COP_{{\text{int}}}$$: Comprehensive coefficient
$F$: Heat transfer area (m^2^)
$f$: The ratio of the summation of high temperature heat exchanger area and the heat recovery heat exchanger area to the total heat exchanger area
$f_{0}$: The ratio of heat recovery heat exchanger area to the summation of high temperature heat exchanger area and the heat recovery heat exchanger area
$K$: Heat transfer coefficient (kW K^−^^1^ m^−^^2^)
$n$: Heat recovery ratio
$P$: Input power (kW)
$Q$: The rate of heat transfer (kW)
$R$: Refrigerating capacity (kW)
$$T$$: Absolute temperature (K)
$U$: Heat transfer coefficient (kW K^-1^)

## Greek letter

$\Phi$: Internal irreversible factor
$$\varepsilon$$: Refrigeration coefficient
$\varepsilon_{{\text{R}}}$: Heat recovery coefficient
$\eta_{{}}$: Effectiveness of heat exchanger

## Subscripts

${\text{COP}}_{{{\text{int}}}}$: Corresponding to comprehensive coefficient
*f*_*−opt*_: Optimum value
*h*: Parameters of the high temperature heat reservoir
$H$: High temperature side
$HC$: Temperatures of the working fluid in high temperature side
$Hi$: Inlet temperature of the high temperature reservoir
$Ho$: Outlet temperature of the high temperature reservoir
*i*: Indoor environment
*l*: Parameters of low temperature heat reservoir
$L$: Low temperature side
$LC$: Temperatures of the working fluid in low temperature side
$Li$: Inlet temperature of the low temperature reservoir
$Lo$: Outlet temperature of the low temperature reservoir
*o*: Outdoor environment
$$P$$: Input power
*r*: Parameters of the heat recovery reservoir
$R$: Heat recovery side
$Ri$: Inlet temperature of the heat recovery reservoir
$Ro$: Outlet temperature of the heat recovery reservoir
$\varepsilon$: Corresponding to refrigeration coefficient
$\varepsilon_{{\text{R}}}$: Corresponding to heat recovery coefficient
$\eta_{\Pi }$: The exergy efficiency
